# Promotion of Zinc Tablets with ORS through Child Health Weeks Improves Caregiver Knowledge, Attitudes, and Practice on Treatment of Diarrhoea in Nigeria

**Published:** 2015-03

**Authors:** Jacqueline K. Kung'u, Olumuyiwa Owolabi, Grace Essien, Francis T. Aminu, Ismael Ngnie-Teta, Lynnette M. Neufeld

**Affiliations:** ^1^Micronutrient Initiative, Regional Office-Africa; ^2^Nutrition Section, Biochemistry Department, Ahmadu Bello University, Zaria, Nigeria; ^3^Independent Consultant (formerly with Micronutrient Initiative); ^4^Global Alliance for Improved Nutrition (formerly with Micronutrient Initiative); ^5^UNICEF Guinea (formerly Micronutrient Initiative)

**Keywords:** Child Health Week, Diarrhoea, ORS, Zinc, Nigeria

## Abstract

We examined whether the Maternal, Newborn and Child Health Weeks (MNCHW) in Nigeria would present an opportunity to raise awareness of and demand for the use of zinc and ORS in the treatment for diarrhoea, guided by a conceptual framework designed to assess three theoretical underpinnings (characteristics and performance standard of the health workers, potential reach, and intensity of the intervention), along the impact pathway. Zinc and ORS with education for their appropriate use during the next diarrhoeal episode were delivered as part of the November 2010 and May 2011 MNCHW. On the day of but before participating in MNCHW activities, semi-structured interviews were used for collecting information on knowledge, attitudes, and practice (KAP) relating to diarrhoea from 602 caregivers with children aged less than five years. Forty-eight health workers were also interviewed. Nearly all health workers (98%) correctly mentioned the dosage of zinc while only 58% correctly stated the preparation of ORS. The proportion of caregivers with knowledge on the treatment for diarrhoea increased from 46.4% in November 2010 pre-MNCHW to 71.3% in May 2011 pre-MNCHW interviews (p<0.001). More caregivers correctly mentioned the dosage of zinc (80.9%) and stated the preparation of ORS (88.8%) in the November 2010 exit interview immediately after the MNCHW encounter compared to the levels a few months later in the home follow-up visit (53.1% and 37.4% respectively). After attending both rounds of November 2010 and May 2011 MNCHW, caregivers’ knowledge on the treatment of diarrhoea increased seven times compared to the caregivers who attended the May 2011 MNCHW only (OR=7.0, p<0.001). Additionally, caregivers were 40% less likely to seek advice outside the home in the treatment for diarrhoea if they had attended both the MNCHWs than if they had attended the May 2011 MNCHW only (OR=0.6, p<0.029). We conclude that providing opportunities for caregivers to receive a sample of zinc and ORS and to learn about its use in the treatment for diarrhoea, from trained health workers during MNCHW, has the potential to increase KAP relating to the use of zinc and ORS in the treatment for diarrhoea and for future diarrhoeal episodes.

## INTRODUCTION

Worldwide, diarrhoea is the second leading cause of death in children and is a leading cause of malnutrition and mortality in children aged less than five years in developing countries (1,2). On average, under-five children experience 2.9 episodes of diarrhoea per year in developing countries (3). Zinc deficiency is also prevalent among young children in developing countries that have a poor diet and high exposure to gastrointestinal parasites (4). It is associated with a dysfunctional immune system, growth retardation, and a high risk of morbidities, such as diarrhoea and acute respiratory infections and, subsequently, is responsible for 14% of diarrhoeal deaths among children between 6 months and 5 years of age in Latin America, Africa, and Asia (5–7).

Studies show that supplemental zinc, when combined with low-osmolarity oral rehydration salts solution (ORS), provides therapeutic benefits in diarrhoea. Zinc supplementation, combined with ORS, reduces the duration and severity of acute and persistent diarrhoea (7–10); one study has shown a reduced incidence of diarrhoea in the 2-3 months following a diarrhoeal episode (11). Based on available evidence, WHO and UNICEF recommend zinc (daily 20 mg zinc supplements for 10-14 days for children with acute diarrhoea and 10 mg per day for infants below six months of age) with ORS in the treatment for diarrhoea (12). Although the international guidelines exist and a number of developing countries, including Nigeria, have added zinc treatment to their national policy on the treatment for diarrhoea, most countries have not yet implemented the programmes (13).

The need to increase care-seeking and to undertake effective programmes for managing diarrhoea in Nigeria is great. According to the 2008 Nigeria Demographic and Health Survey, 10.1% of children aged less than five years had a diarrhoeal episode in the 2 weeks prior to the survey, 42% of whom were taken to a healthcare clinic for advice or treatment, and 29.2% did not receive any treatment (14). Second, there is a high prevalence of zinc deficiency in Nigeria. National prevalence is estimated at 20%, slightly higher in rural (26%) than urban areas (17%) (15). Lastly, some studies in Nigeria have reported that there exists a gap in the knowledge, attitudes, and practice (KAP) in relation to appropriate treatment practices for diarrhoea among caregivers (16,17). Some progress to incorporate zinc in the treatment for diarrhoea, using the primary healthcare (PHC) workers as the delivery channel, has been made. However, the use of PHC centres is low in many parts of Nigeria (14). Given that introduction of zinc and ORS in the treatment for diarrhoea is a new intervention in Nigeria, there is an urgent need to scale up educational and promotional activities to create demand for this among healthcare providers and caregivers.

Child Health Weeks provide a unique opportunity to raise awareness of and create demand for new and novel health interventions. Most Child Health Weeks are characterized by delivery of an integrated package of child and maternal survival interventions and social mobilization activities of high priority. These have been shown to improve coverage of periodic interventions, such as measles immunization, vitamin A supplementation, deworming, and access to insecticide-treated nets. These events may be an appropriate platform for the distribution of zinc and ORS (18,19). The characteristics and performance standard of the health workers, the ‘potential reach’, and the ‘intensity of the intervention’ using the Child Health Week as a platform to create demand have not been evaluated. ‘Potential reach’ is defined as optimal service delivery, and behaviour change interventions reach all the identified beneficiaries of the programme; and the ‘intensity of the intervention’ is defined in terms of the number of MNCHW contacts the caregiver had (i.e. one MNCHW contact was defined as low intensity while having two MNCHW contacts was defined as high intensity). We, therefore, designed this operational research study to explore whether the MNCHW would present an opportunity to raise awareness of and demand for zinc and ORS among women in Osun State, Nigeria, in the treatment for diarrhoea.

## MATERIALS AND METHODS

### Study site

The study was conducted in Osun State in South Western Nigeria. Osun State was one of the 4 states where the Micronutrient Initiative supported the vitamin A supplementation programme. Osun State is made up of 30 local government areas (LGAs) and 3 senatorial districts. We purposively selected 3 LGAs—one from each senatorial district, based on their high prevalence of diarrhoea. This was expected to increase our probability of obtaining caregivers who had under-five children with diarrhoeal episode in the last 3 months prior to the MNCHW. We then randomly selected half of the health clinics in each LGA for the interviews.

### Study design

#### The intervention

As part of a public health strategy in Osun State of Nigeria, zinc and ORS were distributed via the public health clinic services. However, since the use of clinic services was low, the decision was made to test whether the provision of and training relating to the use of zinc and ORS at the MNCHW, regardless of whether the child had diarrhoea at that moment, could raise awareness of and stimulate demand for zinc and ORS for future diarrhoeal episodes. Therefore, a take-home diarrhoea management kit intended for use in the next diarrhoeal episode, consisting of one treatment course of zinc tablets (10 tablets) bundled with ORS (2 sachets), was provided to all caregivers with children aged less than 5 years during the MNCHW in November 2010. A communication strategy was also implemented, which included on-site counselling on the treatment for diarrhoea as well as suitable printed material in the take-home kit in Yoruba, with pictorial illustrations on how to utilize zinc and ORS, when to take the child to the health clinic, and how to prevent future diarrhoea.

#### Evaluating the effect of intervention

To examine the effectiveness of the pre-MNCHW education campaign and counselling provided during the MNCHW on the treatment of diarrhoea for the caregivers on changes in KAP, we defined three parameters as the theoretical framework for our analyses that would explain the pathway from intervention to effect. First, we evaluated the ‘characteristics and performance standard of the health workers’ by conducting an interview among health workers to determine their characteristics, experience, training, and extent to which healthcare providers identify zinc and ORS as an appropriate treatment for diarrhoea.

Second, the ‘potential reach’ was evaluated by measuring changes in KAP relating to the treatment for diarrhoea in pre-MNCHW and post-intervention. Post-intervention ‘potential reach’ was examined immediately after the MNCHW contact at the exit interview and in the time-interval between the November 2010 and May 2011 MNCHW (5 months). During the November 2010 and May 2011 MNCHW, sets of semi-structured questionnaire with both closed- and open-ended questions were administered to selected caregivers prior to their encounter with healthcare providers. These are two cross-sectional surveys with caregivers conducted in November 2010 and May 2011. Comparison between the two time-points shows us what has changed over time in comparison with the previous survey and not changed over time on the same individuals. The objective of these two pre-MNCHW interviews was to assess the caregiver's current KAP relating to diarrhoea and its treatment. We conducted exit interviews of a small subset of caregivers upon leaving the MNCHW to assess how well the caregiver recalled information relating to zinc and ORS immediately after the MNCHW. We further assessed how well the caregiver recalled information relating to zinc and ORS five months after the MNCHW. The period of five months was selected because it was the longest time within the time-interval between the November 2010 and May 2011 MNCHW when we could conduct the home follow-up interviews. We used the health clinic register to identify caregivers who had come for the treatment for diarrhoea after the November 2010 MNCHW and interviewed them at home.

Third and last, we have defined the ‘intensity of the intervention’ operationally in terms of the number of MNCHW contacts the caregiver had (i.e. one MNCHW contact was defined as low intensity while having two MNCHW contacts was defined as high intensity). The ‘intensity of the intervention’ was evaluated by determining whether participation in the first MNCHW predicts KAP at the second MNCHW.

### Estimation of sample-size and sampling procedure

#### Health workers’ interview

We evaluated the characteristics and performance standard of the health workers by addressing the first question, using provider interviews: To what extent do healthcare providers identify zinc and ORS as an appropriate treatment for diarrhoea? All health workers at the 60 participating health clinics that were providing zinc for the treatment of diarrhoea cases were eligible for the interview. A total of 48 health workers were available for the interview. This interview was conducted 5 months after the November 2010 MNCHW; at the same time, the home follow-up visit interviews were conducted.

### Pre-MNCHW interview on KAP

The November 2010 pre-MNCHW interview was conducted before distribution of the take-home kits while the May 2011 pre-MNCHW was conducted after the take-home kits had been purposefully made available in the health facilities but before encounter with the health worker at the MNCHW.

The sample-size for the pre-MNCHW interviews was calculated considering knowledge on the treatment for diarrhoea as the primary outcome. At baseline, the proportion of caregivers with ‘adequate knowledge’ on the treatment for diarrhoea was expected to be 40% (14); this is the percentage of children with diarrhoea for whom advice or treatment was sought from a health facility or care provider. We estimated that a sample-size of 303 caregivers was sufficient to detect differences of 10% in knowledge on the treatment for diarrhoea in the baseline and endline pre-MNCHW interview, assuming a desired precision of 5% within the study area. We used a modest design effect of 2, resulting in a sample-size of 606 caregivers.

To achieve this estimate, a minimum of 10 caregivers with under-five children attending the MNCHW in the participating 60 health clinics were randomly selected by systematic sampling in which every *k*th caregiver was selected, where *k*=number of caregivers visiting the health clinics÷10 (the number of caregivers interviewed at each health clinic). The pre-MNCHW interviews obtained data, using a semi-structured questionnaire on current KAP relating to diarrhoea and its treatment and evaluated both ‘potential reach’ and ‘intensity of the intervention’ by addressing the following two operational research questions respectively: (i) what is KAP relating to the treatment for diarrhoea generally and specifically, using zinc and ORS before its delivery at the MNCHW and implementation of a communication strategy and at a subsequent MNCHW six months later? and (ii) did participation in the first MNCHW predict KAP at the second MNCHW?

#### Exit interviews and home follow-up visit

To determine how well caregivers recalled information related to zinc and ORS when visiting health clinics 5 months after the MNCHW compared to immediately after it, we interviewed a small subset of caregivers upon leaving the MNCHW and made home follow-up visit for others.

The exit interview and the home follow-up visit were conducted to further evaluate the ‘potential reach’. We specifically addressed how well caregivers recalled information relating to zinc and ORS immediately after the MNCHW and in the time-interval between the November 2010 and May 2011 MNCHW (5 months). We conducted the exit interviews on a small subset of caregivers immediately upon leaving the MNCHW in November 2010 and May 2011.

We then conducted home follow-up visits to assess information recall and perceptions towards zinc and ORS among women who had sought treatment for diarrhoea at the health clinic. All caregivers who had sought treatment for diarrhoea in the time-interval between the November 2010 and May 2011 MNCHW from the participating 60 health clinics were eligible to participate. One HF was selected per ward in at least six wards within the LGA. We then randomly selected 10 caregivers from the list of caregivers who had attended each participating health clinic since the November 2010 MNCHW, resulting in a total of 180 caregivers.

### Informed consent and ethical clearance

Ethical clearance for the study was obtained from the Ministry of Health, Osun State. Community leaders were also informed of the study objectives and procedures to obtain collective consent. The objective, methods, risks, and benefits of participation were explained to the participants who were then given a chance to ask questions about the study and, if in agreement to participate, were asked for their verbal consent. The verbal consent was similar for all stages of the interview.

### Data-entry and analysis

During the interviews, sets of filled questionnaire were collected and reviewed by research assistants for completeness and errors in the field. The data were then entered into Excel (Microsoft Incorporation, 2010) and analyzed using PASW software (version 18: SPSS). Open-ended questions were systematically coded for analysis. KAP was operationalized as follows: (i) *knowledge*: we used messages about treatment for diarrhoea heard or seen in the past 3 months preceding the interviews; (ii) *attitudes*: the response from caregivers when they were asked whether they believed statements on efficacy and effective delivery systems of zinc for the treatment of diarrhoea; and (iii) *practices*: seeking care in the treatment for diarrhoea at the health clinic. Chi-square test was used in order to compare proportions at baseline (November 2010 MNCHW) and endline (May 2011 MNCHW), with p<0.05 considered significant. Binary logistic regression was used in order to calculate the odds ratio of a caregiver having knowledge on the treatment for diarrhoea and a caregiver seeking advice outside the home on the treatment for diarrhoea in May 2011. To answer the operational research question whether participation in the first MNCHW predicted KAP at the 2nd MNCHW, we ran a binary logistic regression model with the May 2011 pre-MNCHW dataset, using knowledge on the treatment for diarrhoea as the dependent variable. We adjusted for LGA, education status, age, number of under-five children, whether or not the caregiver had attended the November 2010 MNCHW, and diarrhoea in the last three months. We also ran a similar binary logistic regression using caregiver seeking advice outside the home on the treatment for diarrhoea as the dependent variable.

## RESULTS

### Characteristics of caregivers and performance standard of the health workers

The mean age of the caregivers interviewed during both the pre-MNCHW interviews was 27 years (Table 1). Most of the caregivers had attained higher than secondary school education (>60%).

**Table 1.** Characteristics of the caregivers interviewed during the November 2010 and May 2011 pre-MNCHW

Variable	November 2010 pre-MNCHW interview (N=593)	May 2011 pre-MNCHW interview (N=602)
Local Government Area (LGA)	No. (%)	No. (%)
Boripe LGA	200 (33.7)	200 (33.2)
IFE North LGA	203 (34.2)	202 (33.6)
Ola Oluwa LGA	190 (32.0)	200 (33.2)
Mean age (years)	Mean±SD	Mean±SD
	27.22±6.02	27.29±5.40
Education	No. (%)	No. (%)
No education	43 (7.3)	24 (4.0)
Primary education	174 (29.3)	114 (18.9)
≥Secondary education	376 (63.4)	464 (77.1)
Mean number of under-five children	Mean±SD	Mean±SD
	1.77±0.90	1.64±0.77

We evaluated the performance standard of the health workers by assessing the extent to which healthcare providers identified zinc and ORS as an appropriate treatment strategy for diarrhoea. All the health workers interviewed had been trained on the management of diarrhoea using zinc and ORS and had been involved in the treatment of diarrhoeal children aged less than five years. Half (50%) of them were community health extension workers, and close to half (43.8%) had served as health workers for 10-19 years. The mean service length was 15.3±8.6 years (Table 2). When asked to list the drugs they usually used for under-five children with diarrhoea, 98% listed zinc and ORS and correctly mentioned the dose of zinc. However, only about 60% of the health workers correctly indicated that 1 sachet of ORS was to be dissolved in 1 litre of water (Table 2). These health workers did not differ significantly from those who gave the wrong answer in terms of their cadre (p=0.536), service length (p=0.611), or the time when they were trained (p=0.272) (data not shown).

### Evaluation of the ‘potential reach’ of the intervention

#### KAP relating to treatment for diarrhoea using zinc and ORS

We found that the proportion of caregivers with knowledge on the treatment for diarrhoea increased from 46.4% in the November 2010 pre-MNCHW interview to 71.3% in the May 2011 pre-MNCHW interview (p<0.001); messages about treatment for diarrhoea heard or seen in the past 3 months preceding the interview were used as a proxy for knowledge on the treatment for diarrhoea. In both instances, community health workers were reported as the primary sources of knowledge on diarrhoea. As a proxy for attitudes, caregivers were asked whether they believed statements on the use of zinc in the treatment for diarrhoea. In November 2010, about half of the caregivers (ranging between 43.5% and 54.5% for different statements) believed these statements while, in May 2011, over two-thirds of the caregivers (69.3%-79.7%) believed these statements (p<0.001 for all comparisons). Lastly, healthcare-seeking behaviour among caregivers was reported as a proxy for practices on the treatment for diarrhoea. Majority of caregivers whose children ever had diarrhoea sought advice from someone outside the home (67.9% in November 2010 and 79.7% in May 2011) (p<0.001) (Table 3). The places where and who the caregivers sought advice from were health facilities and chemists.

#### Caregivers recall of information related to zinc and ORS

Exit interviews showed that big proportions of respondents knew about the correct dosage of zinc and the preparation of ORS. However, home-visit showed that comparatively a lower proportion of respondents was able to mention the correct dosages of zinc and preparation of ORS compared to exit interviews (p<0.001). The number of caregivers who had seen or heard message on the treatment for diarrhoea increased at the May 2011 MNCHW exit interview and the home follow-up visit compared to the November 2010 MNCHW exit interview (p<0.001), which was expected (Table 4).

**Table 2.** Characteristics of health workers and appropriate information on the treatment for diarrhoea^1^

Cadre of health workers interviewed	N=48
Junior community health extension workers	6 (12.5)
Community health extension workers	24 (50.0)
Nurse	4 (8.3)
Midwife	3 (6.3)
Community health officer	8 (16.7)
Health assistant	3 (6.3)
Mean±SD service length of the health workers interviewed	15.3±8.6
Trained <10 years	14 (29.2)
Trained 10-19 years	21 (43.8)
Trained ≥20 years	13 (27.1)
When trained	
Before May 2011 MNCHW	20 (41.7)
Before November 2010 MNCHW	28 (58.3)
Drugs the health workers used for treating diarrhoea^2^	
Zinc	47 (97.9)
ORS	47 (97.9)
Antibiotics	15 (31.3)
Others	3 (6.3)
Number of health workers who correctly mentioned the dosage of zinc^3^	47 (97.9)
Number of health workers who correctly stated the preparation of ORS^4^	28 (58.3)
Effect of zinc as recalled by health workers^2^	
Reduce duration of diarrhoea	44 (91.7)
Reduce severity of diarrhoea	35 (72.9)
Prevent recurrence of diarrhoea	17 (35.4)

^1^Values are number (%) or mean±SD

^2^Multiple responses

^3^<6 months=½ tablet/day × 10 days; 6-59 months=1 tablet/day × 10 days

^4^Dissolve 1 sachet of ORS in 1 litre of water

### Evaluation of the ‘intensity of intervention’ by assessing whether participation in the first MNCHW predicts KAP at the second MNCHW

After attending both rounds of the November 2010 and May 2011 MNCHW, caregivers’ knowledge on the treatment for diarrhoea increased seven times compared to the caregivers who did not attend the November 2010 MNCHW (OR=7.0, p<0.001) (Table 5). Additionally, we found that the likelihood of caregivers seeking advice outside the home in treatment for diarrhoea was reduced by 40% if the caregiver had attended both MNCHW compared to if they had only attended the May 2011 MNCHW only (OR=0.6, p=0.029) (Table 6).

## DISCUSSION

The use of zinc in the management of childhood diarrhoea is recommended by WHO and UNICEF (12). If done effectively, scale-up of zinc and ORS will greatly reduce the burden of preventable deaths caused by diarrhoea. However, two prerequisites necessary in ensuring effective scale-up are: ensuring wide availability of these commodities within the public and private health systems and demand among providers and caregivers for the use of zinc and ORS in the treatment for diarrhoea (20). Although previously-conducted randomized trials have provided us with the evidence of efficacy, these do not provide the much-needed evidence of how to implement programmes effectively. We took advantage of Nigeria's plans to implement zinc through the public health clinics and the ongoing MNCHW to test the latter as a route to raise awareness and demand for the use of zinc and ORS for the treatment in future diarrhoeal episodes. Experiences from various countries show that coverage of child survival interventions are improved through integrated packages delivered through MNCHW (19,21) but this has not been adequately tested for interventions that would be used at home for future health events. Although many countries are moving away from campaign-style health services in countries, like Nigeria, where the reach of the health system is extremely limited, they provide an important channel to increase demand for those services.

**Table 3.** KAP on the treatment for diarrhoea among caregivers interviewed during the November 2010 and May 2011 pre-MNCHW^1^

Variable	November 2010 pre-MNCHW interview (N=593)	May 2011 pre-MNCHW interview (N=602)	p value in χ^2^-test
Knowledge on the treatment for diarrhoea^2^	275 (46.4)	429 (71.3)	<0.001
Source of knowledge on diarrhoea^3^			
Radio	98 (16.5)	67 (11.1)	0.007
Television	30 (5.1)	14 (2.3)	0.012
Education session/Health talk	50 (8.4)	95 (15.8)	<0.001
Community dialogue session	14 (2.4)	26 (4.3)	0.060
Community health worker	146 (24.6)	278 (46.2)	<0.001
Neighbour/Friend/Relative	65 (11.0)	113 (18.8)	<0.001
Banner/Poster/Newspaper	7 (1.2)	1 (0.2)	0.032
Attitudes on using zinc for the treatment for diarrhoea^4^			
Zinc reduces the duration of diarrhea	258 (43.5)	480 (79.7)	<0.001
Zinc reduces the severity of diarrhea	261 (44.0)	460 (76.4)	<0.001
Zinc is available in pharmacy/chemist/health centres	229 (38.6)	417 (69.3)	<0.001
Zinc should be taken with ORS/ORT	254 (42.8)	475 (78.9)	<0.001
A complete 10-14 day dose should be administered	259 (43.7)	472 (78.4)	<0.001
Zinc and ORS are an appropriate treatment for diarrhoea	323 (54.5)	459 (76.2)	<0.001
Healthcare-seeking behaviour among caregivers whose children had diarrhoea^5^			
Sought advice from someone outside the home (health facility or chemist)	239/352 (67.9)	382/479 (79.7)	<0.001

^1^Values are number (%)

^2^Messages about treatment for diarrhoea heard or seen in the past 3 months preceding the interview were used as a proxy for knowledge on the treatment for diarrhoea

^3^Multiple responses

^4^Responses from caregivers when asked whether they believed statements on the use of zinc for the treatment for diarrhoea were used as a proxy for attitudes on the treatment for diarrhoea

^5^Healthcare-seeking behaviour among caregivers was used as a proxy for practices on treatment for diarrhoea

Delivery of interventions needs knowledgeable health workers who then have access to refresher training on the key interventions that they deliver. From our study, only just over half of health workers could accurately describe preparation methods for ORS. Those health workers who gave inaccurate information may not have been recently trained on diarrhoea management generally, or specifically on zinc or ORS. Indeed, more formative research is needed to help assess that the introduction of zinc has not negatively impacted the use of ORS and other medicines. Although we evaluated the ‘characteristics and performance standard of the health workers’, one of the weaknesses of this study is that it was not feasible for us to fully assess fidelity of the interventions by the health workers, particularly the quality and replicability of message delivery. It was particularly challenging to evaluate whether the health workers delivered accurate (and similar) messages throughout implementation of the intervention. This could have been very long and intense days’ observations and/or data collection during the delivery of the intervention itself, which was not feasible at the time of this study and in this context.

**Table 4.** Association between MNCHW encounter and recall of information on the treatment for diarrhoea with zinc and ORS^1^

Characteristics	November 2010 MNCHW exit interview (N=89)	p value^4^	Home follow-up visit (N=179)	p value^5^	May 2011 MNCHW exit interview (N=153)	p value^6^
Number of caregivers who correctly mentioned the dosage of zinc^2^	72 (80.9)	p<0.001	95 (53.1)	p<0.001	129 (84.3)	0.4953
Number of caregivers who correctly stated the preparation of ORS^3^	79 (88.8)	p<0.001	67 (37.4)	p<0.001	99 (64.7)	0.0001
Number of caregivers who had seen or heard messages on the treatment for diarrhoea prior to the MNCHW	52 (58.4)	0.1403	121 (67.6)	0.0001	132 (86.3)	p<0.001

^1^Values are number (%)

^2^<6 months=½ tablet/day × 10 days; 6-59 months=1 tablet/day × 10 days

^3^Dissolve 1 sachet of ORS in 1 litre of water

^4^Comparisons between the November 2010 MNCHW exit interview and the home follow-up visit

^5^Comparisons between the home follow-up visit and the May 2011 MNCHW exit interview

^6^Comparisons between the November 2010 MNCHW exit interview and the May 2011 MNCHW exit interview

We evaluated the ‘potential reach’ of the intervention—in this case the extent to which KAP relating to diarrhoea and its treatment change among caregivers after zinc and ORS has been distributed using the platform of the MNCHW and supplies have become available in the healthcare facilities. Our findings show that there was an improvement in KAP between the November 2010 pre-MNCHW interview and the May 2011 pre-MNCHW interview. Specifically, the proportion of caregivers with knowledge on the treatment for diarrhoea increased by 25 percent points between the two MNCHW interviews. The reported source of this knowledge was from zinc promotional activities and the health workers. Take-home diarrhoea management kits were introduced in the PHC for the first time during the November 2010 MNCHW in Osun State. At the same time, zinc-promotional activities were also initiated and continued during the May 2011 MNCHW. It is likely that these promotional activities contributed to a higher proportion of caregivers having knowledge on the treatment for diarrhoea in May 2011. Elsewhere, community mobilization through the mass media has been found to play an important role in increasing caregivers’ knowledge about zinc (22). Attitudes regarding the use of zinc in the treatment for diarrhoea and practices, measured by care-seeking behaviour, also improved between the November 2010 and May 2011 pre-MNCHW interviews.

We could not evaluate whether the choice by the caregiver to visit the health clinic was after the first diarrhoeal episode following the November 2010 MNCHW since we did not have a question during the May 2011 pre-MNCHW asking whether they had used the zinc and ORS that were given in the last MNCHW. Whichever the case, the communication material provided alongside the zinc and ORS was intended to equip the caregivers to recognize the symptoms of diarrhoea, to give appropriate home-care and to decide to go to the health clinic when symptoms warrant. We, therefore, expect that it guided their practices on the treatment for diarrhoea but causality cannot be established in this cross-sectional study. Perhaps one of the interesting findings with regard to exposure to both MNCHWs was that the likelihood of caregivers seeking advice outside the home was reduced. One interpretation for this could be that they already had the take-home diarrhoea management kit provided at the health facility and intended for use in the next diarrhoeal episode, and they used it. Since we did not evaluate whether the caregivers, indeed, used the take-home kit provided, we strongly suggest that future similar studies look into this.

**Table 5.** Logistic regression with knowledge of caregivers on the treatment for diarrhoea as dependent variable

Independent variable	May 2011 pre-MNCHW interview (N=602)			
	Unadjusted odds ratio (95% CI)	p value	Adjusted odds ratio (95% CI)	p value
Local Government Area (LGA)^1^				
Ife North LGA	0.2 (0.1-0.3)	<0.001	0.3 (0.2-0.5)	<0.001
Ola Oluwa LGA	1.4 (0.8-2.4)	0.218	1.1 (0.6-2.0)	0.84
Educational status^2^				
Primary	0.3 (0.6-1.2)	0.086	0.3 (0.1-1.8)	0.20
≥Secondary	0.2 (0.0-0.9)	0.034	0.4 (0.1-1.9)	0.23
Age^3^	1.0 (1.0-1.0)	0.838	1.0 (1.0-1.1)	0.36
Number of under-five children^3^	1.7 (1.3-2.2)	<0.001	1.1 (0.8-1.5)	0.60
MNCHW^4^				
Both November 2010 and May 2011	10.0 (6.6-14.9)	<0.001	7.0 (4.5-10.9)	<0.001
Diarrhoea in the last 3 months^5^				
Yes	3.0 (2.0-4.3)	<0.001	2.0 (1.3-3.2)	0.003

^1^LGA reference=Boripe LGA

^2^Educational status reference=No education

^3^Age and number of under-five children are entered in the model as continuous variables

^4^MNCHW reference=Caregivers who attended the May 2011 MNCHW only

^5^Diarrhoea in the last 3 months reference=Caregivers with children who did not have diarrhoea in the last 3 months

**Table 6.** Logistic regression with seeking advice outside home in the treatment for diarrhoea as the dependent variable

Independent variable	May 2011 pre-MNCHW interview (N=602)			
	Unadjusted odds ratio (95% CI)	p value	Adjusted odds ratio (95% CI)	p value
Local Government Area (LGA)^1^				
Ife North LGA	2.6 (1.4-4.7)	0.003	2.0 (1.0-3.8)	0.042
Ola Oluwa LGA	2.7 (1.6-4.7)	<0.001	3.4 (1.9-6.1)	<0.001
Educational status^2^				
Primary	0.9 (0.3-2.7)	0.853	0.9 (0.3-2.8)	0.868
≥Secondary	1.0 (0.4-2.7)	0.969	1.1 (0.4-3.2)	0.844
Age^3^	1.0 (1.0-1.1)	0.392	1.0 (1.0-1.1)	0.461
Number of under-five children^3^	1.0 (0.7-1.3)	0.740	1.0 (0.7-1.4)	0.981
MNCHW^4^				
Both November 2010 and May 2011	0.6 (0.4-1.0)	0.042	0.6 (0.3-0.9)	0.029
Diarrhoea in the last 3 months^5^				
Yes	0.8 (0.5-1.3)	0.437	0.7 (0.4-1.1)	0.136

^1^LGA reference=Boripe LGA

^2^Education status reference=No education

^3^Age and number of under-five children are entered in the model as continuous variables

^4^MNCHW reference=Caregivers who attended the May 2011 MNCHW only

^5^Diarrhoea in the last 3 months reference=Caregivers with children who did not have diarrhoea in the last 3 months

We also found that participation in the first MNCHW strongly predicted KAP on the treatment for diarrhoea in the second MNCHW. It is clear from these findings that the multiple MNCHW contacts or the high-intensity intervention was a strong determinant of KAP of the caregivers on the treatment for diarrhoea. Besides the number of MNCHW contacts, other parameters that may influence the ‘intensity of the intervention’ are: length of exposure, duration, and number of communication channels. We do not believe there was significant variation in these other parameters among the caregivers in Osun State.

Additionally, more caregivers correctly mentioned the dosage of zinc and stated the preparation of ORS immediately after the MNCHW compared to a few months later. It is possible that caregivers could have simply forgotten, 5 months after the MNCHW, the health messages communicated to them. To address this, there is need for creating opportunities for the caregivers to meet with health personnel for continuous reinforcing of health information. It is also likely that the healthcare providers did not continue to communicate the messages on the treatment for diarrhoea with zinc and ORS after the November 2010 MNCHW routinely. This could be as a result of high turnover among the trained health workers, lack of training on the use of zinc and ORS for the treatment of diarrhoea for all health workers, or inadequately motivated health workers to perform their duties. Health workers need to receive regular training to ensure accuracy and consistency in the health messages that they communicate at the health clinics.

### Conclusions

With this operations research study using an adequacy impact evaluation design, we have demonstrated the importance of the three theoretical underpinnings of this study. We evaluated the ‘potential reach’ of the intervention using the MNCHW platform and have established that it, indeed, does present an opportunity to raise awareness and improve practice relating to the treatment of diarrhoea with zinc and ORS. We also evaluated the ‘intensity of the intervention’ and the ‘characteristics and performance standard of the health workers’ and have also established through this study that providing multiple opportunities for caregivers to meet with competent health workers in settings where access to routine services is extremely limited will reinforce the health messages necessary to increase demand for essential nutrition interventions. Despite the inability of the study design to causally link programme activities to observed changes, the results obtained provide information necessary to make decisions on the existing and new programmes.

## ACKNOWLEDGEMENTS

The authors acknowledge Christa Fischer-Walker, Alison Greig and Abdulazziz Adish for technical assistance, especially during conception of the study; Prof I.O. Akinyele and Bimbo Adesanmi of Food Basket Foundation International for their role in developing the communication material, training of health workers, bundling and distribution of the diarrhoea management kit; and Oluloto E. Amuwa, Quadri J. Akorede and Tosin Leshi, the research assistants who helped in the supervision of the field data-collection exercise. The work of the Micronutrient Initiative is supported by the Government of Canada through the Department of Foreign Affairs, Trade and Development Canada, and other generous donors.

**Conflict of interest:** Authors declare no competing interests.
